# A systematic review of peptide-based serological tests for the diagnosis of leishmaniasis[Fn FN1]

**DOI:** 10.1051/parasite/2023011

**Published:** 2023-03-31

**Authors:** Julie Pagniez, Elodie Petitdidier, Oriana Parra-Zuleta, Joana Pissarra, Rachel Bras-Gonçalves

**Affiliations:** UMR177 INTERTRYP 911 avenue Agropolis B.P. 64501 34394 Montpellier France

## Abstract

Serological methods should meet the needs of leishmaniasis diagnosis due to their high sensitivity and specificity, economical and adaptable rapid diagnostic test format, and ease of use. Currently, the performances of serological diagnostic tests, despite improvements with recombinant proteins, vary greatly depending on the clinical form of leishmaniasis and the endemic area. Peptide-based serological tests are promising as they could compensate for antigenic variability and improve performance, independently of *Leishmania* species and subspecies circulating in the endemic areas. The objective of this systematic review was to inventory all studies published from 2002 to 2022 that evaluate synthetic peptides for serological diagnosis of human leishmaniases and also to highlight the performance (e.g., sensitivity and specificity) of each peptide reported in these studies. All clinical forms of leishmaniasis, visceral and tegumentary, and all *Leishmania* species responsible for these diseases were considered. Following PRISMA statement recommendations, 1,405 studies were identified but only 22 articles met the selection criteria and were included in this systematic review. These original research articles described 77 different peptides, of which several have promising performance for visceral or tegumentary leishmaniasis diagnosis. This review highlights the importance of and growing interest in synthetic peptides used for serological diagnosis of leishmaniases, and their performances compared to some widely used tests with recombinant proteins.

## Abstract (fr)

D’une sensibilité et d’une spécificité élevées, faciles à réaliser, économiques et adaptables à un format de test de diagnostic rapide, les méthodes sérologiques devraient répondre aux besoins du diagnostic de la leishmaniose. Actuellement, les performances des tests de diagnostic sérologique, malgré des améliorations avec les protéines recombinantes, varient fortement selon la forme clinique de la leishmaniose et les zones d’endémie. Les tests sérologiques à base de peptides sont prometteurs car ils pourraient compenser la variabilité antigénique et améliorer les performances, indépendamment des espèces et sous-espèces de *Leishmania* circulant dans les zones endémiques. L’objectif de cette revue systématique était d’inventorier toutes les études publiées de 2002 à 2022 qui évaluent les peptides synthétiques pour le diagnostic sérologique des leishmanioses humaines et également de mettre en évidence les performances (dont la sensibilité et la spécificité) de chaque peptide rapporté dans ces études. Toutes les formes cliniques de leishmanioses, viscérales et tégumentaires, et toutes les espèces de *Leishmania* responsables de ces maladies ont été considérées. Suite aux recommandations de la déclaration PRISMA, 1405 études ont été identifiées mais seuls 22 articles répondaient aux critères de sélection et ont été inclus dans cette revue systématique. Ces articles de recherche originaux décrivent 77 peptides différents, dont plusieurs sont prometteurs pour le diagnostic de la leishmaniose viscérale ou tégumentaire. Cette revue met en évidence l’importance et l’intérêt croissant accordés aux peptides synthétiques utilisés pour le diagnostic sérologique des leishmanioses, et leurs performances par rapport à certains tests largement utilisés avec des protéines recombinantes.

## Introduction

Neglected tropical diseases (NTDs) affect more than one billion people worldwide, largely in rural areas of low-income countries [80]. Among NTDs, leishmaniases are considered a major global public health problem with over one billion people at risk of infection living in 98 endemic countries and territories on five continents, and almost 1.3 million new cases reported during the last five years (2015–2020) [77, 81].

Leishmaniases are a group of vector-borne diseases caused by parasites of the genus *Leishmania* which present different clinical forms: visceral (VL) and tegumentary leishmaniasis (TL) that includes cutaneous (CL), and mucosal or mucocutaneous (ML) leishmaniasis. The most common forms are CL, which causes skin sores, and VL, which affects several internal organs, usually the spleen, liver, and bone marrow and which is fatal if not treated. To sustainably fight leishmaniases, control strategies should include early biological diagnosis, and active case detection, which will improve clinical diagnosis, treatment and follow-up, and reduce transmission [21, 78, 79].

To date, there is no gold standard available for the diagnosis of active leishmaniasis. A combination of tests (composite reference standards) is required to achieve the accurate diagnosis of leishmaniases. Three major methods are routinely used: parasitological examination (direct demonstration of parasites in Giemsa-stained smears by microscopic observation or parasite culture), molecular tests (polymerase chain reaction (PCR) technique targeting *Leishmania* DNA), and serology (indirect immunofluorescence (IFA), direct agglutination test (DAT), enzyme linked immunosorbent assay (ELISA), immunochromatographic test (ICT) also known as lateral flow test) [3, 52]. At the point of care, serological tests are widely used because they do not require well-equipped laboratories and experienced staff compared to parasitological or molecular techniques. So far, serological diagnostic tests were developed with soluble antigens or whole-cell lysates. These tests had variable sensitivity depending on the antigen used and low specificity due to cross-reactivity with other pathogens present in endemic areas, such as *Trypanosoma cruzi*, the parasite responsible for Chagas disease [25, 39, 71]. To improve the sensitivity and specificity of the immunodiagnostic tests, several studies have attempted to replace soluble antigens with recombinant proteins. Different antigens such as KMP11, LiP2, K39, K26, A2, KE16 have been used in ELISA and ICT [33] with variable sensitivity and specificity depending on both the kind of recombinant antigens and the areas under study. ICTs based on the rK39 protein (fragment of *Leishmania* (*L.*) *infantum* kinesin-like protein) became the most commonly used tools for VL diagnosis because they are well adapted for field testing and have high specificity. However, WHO reported significant variations in sensitivity among the different manufacturers of rK39-based ICT [76]. Moreover, several studies reported variations in sensitivity depending on the geographical region concerned. Sensitivity was lower in East Africa (36% to 87%), Brazil (61% to 92%), and the Mediterranean basin (52% to 91%), than in the Indian subcontinent (92% to 100%) [5, 6, 20, 32, 34, 50, 64]. For CL and ML diagnosis, serological tests are not commonly used because of their low sensitivity and variable specificity [81]. To increase antibody detection, synthetic peptides containing B-cell epitopes could be used in immunoassays. Their use could improve the accuracy and robustness of diagnostic tests because they are devoid of uninformative or less informative epitopes responsible for background reactions [38]. Their chemical synthesis does not involve handling living organisms and can be standardized for a high level of purity and reproducibility, allowing the production of robust ELISA or ICT [7, 42]. Peptide phage display technology, overlapping peptide libraries covering the entire selected protein sequence or *in silico* B-cell epitope prediction can be used to identify specific peptides [28, 61, 67, 73]. Advances in computational techniques and bioinformatics have also enabled the development of algorithms using several physicochemical, structural, and geometrical aspects of amino acid sequences. Most algorithms are free of charge, can be used online, and provide a fast and scalable way to predict B-cell epitopes *in silico* [65].

To evaluate peptide relevance in the design of new serological diagnostic tests for leishmaniases, we reviewed all studies focusing on peptides for serodiagnosis of visceral and tegumentary leishmaniases published from 2002 to 2022. All clinical forms of human leishmaniasis and all *Leishmania* species responsible for these diseases were considered in this systematic review. We reported the diagnostic performance of peptide-based tests (index tests) against a reference standard.

## Objective

To determine the diagnostic accuracy of peptide-based tests for the diagnosis of active human leishmaniasis.

## Methods

### Eligibility criteria

Original research articles meeting all the following inclusion criteria were eligible:

– Population: any patient with clinical symptoms of leishmaniasis;

– Intervention: diagnosis with index tests defined as any immunological test based on peptides derived from *Leishmania* parasite antigens allowing the detection of anti-*Leishmania* antibodies in human serological samples (serum or plasma). Peptide length must be less than 40 amino acids;

– Comparison: diagnosis with one or several reference standards (parasitological methods, commercial serological tests and/or molecular tests);

– Outcomes: Accurate human leishmaniasis diagnosis. Performance data were described by assessing sensitivity and specificity of the index test. Sensitivity is the probability that the index test result is positive given that leishmaniasis is present, reflecting the ability of the index test to correctly identify individuals with the disease through a positive response. Specificity is the probability that the index test result is negative, given that leishmaniasis is absent, reflecting the ability of the index test to correctly identify individuals without the disease through a negative response [82].

### Information sources

A literature search of four electronic databases was performed in December 2022: PubMed, Web of Science, Worldwide Science and SciELO.

### Search strategy

The search strategy used the following string: “((leishmaniasis*) AND (diagnosis*)) AND (peptide OR epitope)” and filters “(humans))”. Language search was limited to English, Portuguese and Spanish and chronological research from January 2002 to December 2022.

### Selection process

Study eligibility was assessed by two independent investigators (JP and OP) by review of article title and abstract and, when relevant, by text reading. Two other independent investigators (RBG and EP) analyzed pre-selected full texts and confirmed inclusion of studies.

### Data collection process and data items

Data extraction from the full texts included was performed by two independent investigators (JP and OP). The following data were imported to a Microsoft Excel^®^ worksheet: reference, peptide sequence, peptide name given by authors, antigen name, ID UniProt or NCBI accession number of antigen, reference standard, index test, clinical research phase, clinical form evaluated, case origin, and causative *Leishmania* species. The clinical research phases of diagnostic tests described in studies were classified according to Zhou *et al.* [82]. Briefly, Phase I (exploratory) studies are those whose purpose is proof-of-principle of a new diagnostic test in a small number of patients (10–50 archived specimens, retrospective design) with a confirmed disease status versus healthy volunteers. Phase 2 (challenge) studies are those evaluating the diagnostic test with a case-control design in 10–100 individuals in each series. The sampling plan includes patients with a spectrum of targeted disease characteristics versus healthy people or patients with other diseases mimicking the targeted disease. Lastly, Phase 3 (clinical) studies are large-scale prospective studies validating the test in representative sample (hundreds of patients) from the target population (suspected cases).

### Risk of bias assessment and effect measures

The Quality Assessment of Diagnostic Accuracy Studies 2 (QUADAS-2) tool was used to analyze the quality of the included studies and their susceptibility to bias [74]. QUADAS-2 consists of four domains: patient selection, index test, reference standard, and flow and timing. All four domains are judged in terms of the risk of bias and the first three in terms of applicability [74].

A descriptive analysis of quality assessment was performed on the data collected from eligible articles by two independent investigators (JP and OP). Data extraction was checked and any discrepancies were resolved by two others investigators (RBG and EP).

### Synthesis methods

Sensitivity and specificity data were presented according to the clinical form of patient groups used. The reported sensitivity and specificity of peptide-based tests, and their confidence intervals, were plotted using the forest plot function generated by Review Manager version 5.3 (RevMan 5.3) [60]. The predictive values were not specified in all articles, so these were estimated based on the percentage of sensitivity and specificity and the number of samples described by the authors in each group. In some articles, several performances were calculated using different non-case groups (healthy individuals from endemic and non-endemic regions and patients infected by diseases other than leishmaniasis). When possible, the performance of peptide-based tests was reevaluated, including all non-cases in a single control group.

## Results

### Included studies

**Figure 1:**
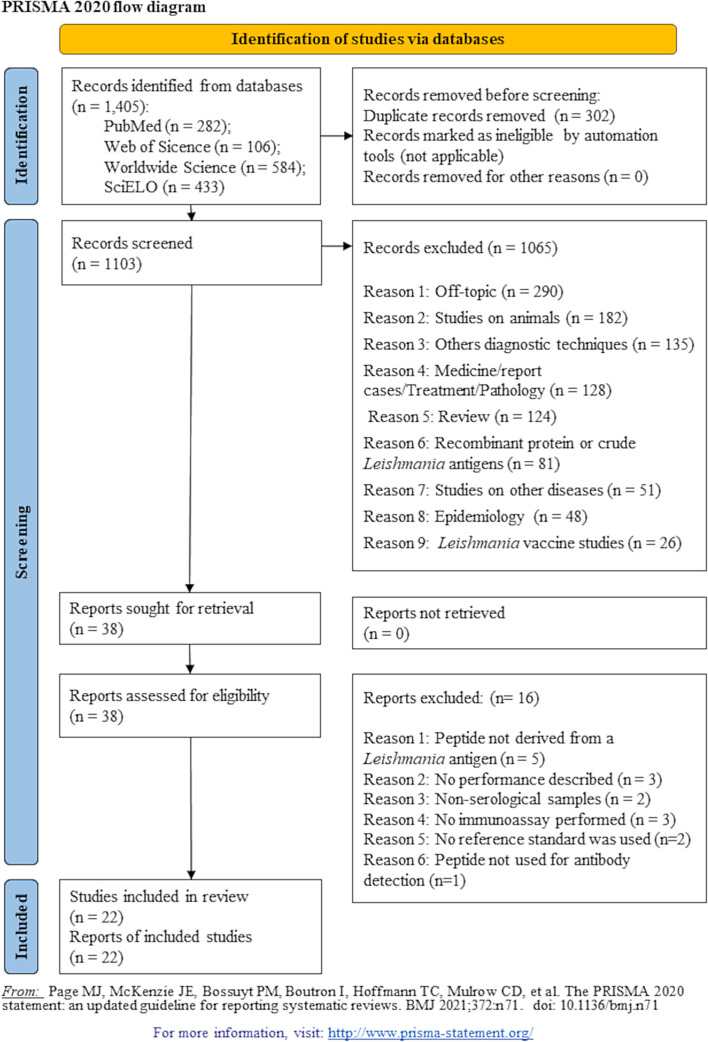
PRISMA Flowchart of the selection steps undertaken in the systematic review.

A total of 1,405 published studies were identified using our search strategy from four databases (Fig. 1). After removing duplicates, 1,103 articles remained. Among them, 1,065 were excluded for several reasons: off-topic (*n* = 290), studies on animals (*n* = 182, including 168 on dogs), other diagnostic techniques (*n* = 135), medicine/report cases/treatment/pathology (*n* = 128), reviews (*n* = 124), recombinant protein or crude *Leishmania* antigens used for antibodies detection (*n* = 81), studies on other diseases (*n* = 51), epidemiology (*n* = 48), and *Leishmania* vaccine studies (*n* = 26). Finally, 38 articles were assessed for eligibility. After a thorough reading of the full text, 16 additional articles were excluded. The reasons for exclusion were the following: peptides were not derived from *Leishmania* antigens (*n* = 5), no performance of the index test was described (*n* = 3), the samples used were not serological samples (*n* = 2), no immunoassay was performed to validate the diagnostic potential of the peptides (*n* = 3), no reference standard was used (*n* = 2), the peptides were not used for antibody detection (*n* = 1). At the end of this screening, 22 studies were included in our systematic review (Supplementary data 1) and the extracted data were compiled in a data collection table (Supplementary data 2).

### Study characteristics

The characteristics of the 22 studies included in this review have been compiled in Table 1. Six studies were classified in clinical research phase I and 16 studies in phase II according to Zhou *et al*. [82], in which the cases were selected from a health service or hospital. To assess the performance of peptide-based tests, three different formats were reported: one study used an ICT format [7], two studies used phage-ELISA [15, 62] and 19 studies used an ELISA technique [10, 11, 16, 17, 26, 27, 29, 35, 37, 40–43, 47, 48, 58, 63, 69, 70]. In these studies using an ELISA method, a marked difference was observed in the amount of peptides used, which ranged from 0.25 μg to 20 μg per well.

**Table 1:** Characteristics of included studies.

Reference	Peptide name given by authors	Reference standard	Index test	Peptide concentration	Clinical research phase	Clinical form evaluated	Case origin	Causative *Leishmania* species
Bremer Hinckel, B.C. *et al*., 2019 [7]	EpQ11	Parasitology, serology (rK39 or rK28)	ICT (cassette)	ND	I	VL	Sudan	ND
			ICT (dipstick)	ND				
Carmelo, E. *et al*., 2002 [10]	23061	Parasitology	ELISA	20 μg/mL	I	CL	Peru	ND
	23063							
	23065							
	23067							
	23069							
	23071							
	23073							
Carvalho, A.M.R.S. *et al*., 2018 [11]	Peptide-1	Molecular technique	ELISA	10 μg/well	II	VL	Brazil	*L. infantum*
	Peptide-2			10 μg/well				
	Peptide-3			10 μg/well				
	Peptide-4			10 μg/well				
	Peptide-5			10 μg/well				
	Peptide-6			10 μg/well				
	Mix I			3.34 μg for each one peptide/well				
	Mix II			1.66 μg for each one/well				
	Mix III			5.0 μg for each one/wel				
	Mix IV			5.0 μg for each one/well				
Costa, L.E. *et al*., 2016 [15]	A10	Parasitology, molecular technique	Phage-ELISA	1.00E + 08 phages/well	II	TL	Brazil	*L. braziliensis*
	B7							
	B10							
	C11							
	C12							
	H7							
Costa, L.E. *et al*., 2017 [16]	B10 Peptide	Molecular technique, serology (Kalazar detect Test)	ELISA	2 μg/well	I	VL	Brazil	*L. infantum*
	C01 Peptide							
Costa, M.M. *et al*., 2012 [17]	47	Parasitology (VL patients); molecular technique (control group)	ELISA	40 μg/mL, 4 μg/well	I	VL	Brazil	ND
	17							
	18							
	19							
	13							
	Mix peptides 13 + 19							
	Mix peptides 18 + 19							
	Mix peptides 13 + 47							
	Mix peptides 17 + 47							
	Mix peptides 19 + 47							
Galvani N.C. *et al*., 2021 [26]	Pept1	Molecular technique (VL patients); serological test (Kalazar detect Rapid test kit, Inbios) (control group)	ELISA	5 μg/well	II	VL	Brazil	*L. infantum*
	Pept2							
	Pept3							
	Pept4							
	Pept5							
	Pept6							
	Pept7							
	Pept8							
Galvani N.C. *et al*., 2022 [27]	Pept1	Parasitology, molecular technique	ELISA	5 μg/well	II	TL	Brazil	*L. braziliensis*
	Pept2							
	Pept3							
	Pept4							
	Pept5							
	Pept6							
	Pept7							
	Pept8							
Gonzales *et al*., 2002 [29]	23083	Parasitology	ELISA	20 μg/mL	I	TL	Peru	ND
	23089							
	23085							
Link, J.S. *et al*., 2017 [35]	P1	Parasitology	ELISA	0.25 μg/well	I	CL	Brazil	*L. braziliensis*
	P2							
	P3							
	Mix P1+P2+P3							
Machado, A.S. *et al*., 2020 [37]	PeptC	Molecular technique (VL patients); serology (control group)	ELISA	10 μg/well	II	VL	Brazil	*L. infantum*
Medeiros *et al*. 2022 [40]	peptide	Parasitology, molecular technique	ELISA	1 μg/well	II	TL	Brazil	*L. braziliensis*
Menezes-Souza, D. *et al*., 2014 [42]	Peptide 1	Parasitology, molecular technique	ELISA	10 μg/well	II	TL	Brazil	*L. braziliensis* (TL); *L. infantum* (VL)
						VL		
	Peptide 2					TL		
						VL		
	Peptide 3					TL		
						VL		
Menezes-Souza, D. *et al*., 2015a [43]	Peptide-1	Parasitology, molecular technique	ELISA	10 μg/well	II	TL	Brazil	ND
						VL		
	Peptide-2					TL		
						VL		
Menezes-Souza, D. *et al*., 2015b [41]	Peptide-1	Parasitology, molecular technique	ELISA	10 μg/well	II	TL	Brazil	ND
						VL		
Oliveira-da-silva, J.A. *et al*., 2020a [47]	Peptide	Molecular technique	ELISA	2 μg/well	II	VL	Brazil	*L. infantum*
Oliveira-da-silva, J.A. *et al*., 2020b [48]	PeptJ	Molecular technique (VL patients); serology (control group)	ELISA	2 μg/well	II	VL	Brazil	*L. infantum*
Ramos F.F. *et al*., 2021 [58]	Pep1	Molecular technique (VL and VL/HIV-coinfeted patients), serological test (Kalazar detect Rapid test kit) (control group)	ELISA	2.5 μg/well	II	VL	Brazil	*L. infantum*
	Pep2			5 μg/well				
	Pep3			5 μg/well				
	Pep4			2.5 μg/well				
	Pep5			1.25 μg/well				
	Pep6			1.25 μg/well				
	Pep7			1.25 μg/well				
	Pep8			2.5 μg/well				
	Pep9			1.25 μg/well				
Salles, B.C.S. *et al*., 2017 [62]	A3	Molecular technique (VL patients); parasitology, molecular technique (TL patients)	Phage-ELISA	1.00E+08 phages/well	II	VL	Brazil	*L. infantum*
	A5							
	A8							
	A11							
	B2							
	B9							
	H11							
	G12							
Salles, B.C.S. *et al*., 2019 [63]	Peptide	Parasitology and molecular technique (TL patitents); serology (control group)	ELISA	1.5 μg/well	II	TL	Brazil	*L. braziliensis*
Vale, D.L. *et al*., 2019 [69]	Peptide	Molecular technique	ELISA	1 μg/well	II	VL	Brazil	*L. infantum*
Vale, D.L. *et al*., 2022 [70]	PepA	Molecular technique	ELISA	5 μg/well	II	TL and VL	Brazil	*L. braziliensis* (TL); *L. infantum* (VL)
	PepB							
	PepC							
	PepD							
	PepE							
	PepF							
	PepG							

A total of 77 different peptide sequences were tested as potential candidates for diagnostic tests (Table 2), ranging from seven to 32 amino acids (Supplementary data 2) in immunoassays, and performance data were collected. In all, 57 peptides were derived from 24 different proteins with identified accessions (Table 2) such as leishmanolysin (gp63) [35], amastin [69], HSP83.1 [42], A2, LACK, NH [17], Histone H1 [10], β-tubulin [16] or tryparedoxin [40, 70]. For the remaining 20 peptides, the protein was not known or was not identified (identification number (ID UniProt (https://www.uniprot.org/) or NCBI accession number (https://www.ncbi.nlm.nih.gov/protein/) are not specified) [15, 17, 58, 62]. Most peptides published in this review were derived from four species of *Leishmania*. A total of 38 peptide sequences were derived of an antigen from *Leishmania infantum*, 33 from *L. braziliensis*, two from *L. donovani*, and one from *L. mexicana.* For only three peptide sequences, the *Leishmania* species were not determined.

**Table 2:** List of peptides described in included studies.

Reference	Peptide sequence	Peptide name given by authors	Antigen name	ID UniProt (protein or gene name) or NCBI accession number of antigen
Bremer Hinckel, B.C. *et al*., 2019 [7]	NIRIHLGDTIRIAPCK	EpQ11	Transitional endoplasmic reticulum ATPase, putative	LDBPK_361420
Carmelo, E. *et al*., 2002 [10]	MFANSSAAAVTAASNSPQRS	23061	Histone H1	AF131892
	SNSPQRSPRPSPKKAAVKKA	23063		
	KKAAAKKAAAKKAAPKKAAP	23065		
	KAAPKRAAPKRAAPKKAAPK	23067		
	APKKAAAKRAAKKSAPKKAV	23069		
	APKKAVKKAVKAAKKAVKKA	23071		
	AVKKAAKKATKRTAKKAAKK	23073		
Carvalho, A.M.R.S. *et al*., 2018 [11]	SGAPRANNSGDASA	Peptide-1	Stabilization of polarity axis, putative	LINJ_30_2730
	GLSGEGSPASPEPRLAGGGGGADTQSTT	Peptide-2		
	DGKPKENQKTARES	Peptide-3	Hypothetical protein, conserved	LINJ_32_0280
	VADSGSASSEDGGSAKP	Peptide-4		
	PRKADPNDTTPQ	Peptide-5	MRP1	LINJ_27_0980
	GDSPPSDSPQNNQDRNRNQN	Peptide-6		
	Mix I peptides 2+3+6	Mix I	Mix	Mix
	Mix II peptides 1+2+3+4+5+6	Mix II	Mix	Mix
	Mix III peptides 2+6	Mix III	Mix	Mix
	Mix IV peptides 3+6	Mix IV	Mix	Mix
Costa, L.E. *et al*., 2016 [15]	ASFLKNR	A10	ND	ND
	SSPFLFS	B7		
	RSMEIDR	B10		
	LEKVFSP	C11		
	KFTLKAR	C12		
	MKFTLNA	H7		
Costa, L.E. *et al*., 2017 [16]	LSFPFPG	B10 Peptide	β-tubulin	XP_001468164.1
	FTSFSPY	C01 Peptide		
Costa, M.M. *et al*., 2012 [17]	VGPQSVGPLSVGPQSVGPLS	47	A2 (amastigote stage-specific S antigen)	ND
	TPAVQKRVKEVGTKP	17	NH (Nucleoside hydrolase)	ND
	TTVVGNQTLEKVT	18		
	VVSTSRDGTAISWK	19	LACK (*Leishmania* analogue of the receptor kinase C)	ND
	ESTTAAKMSAEQDRESTRATLE	13	K39 (putative kinesin 39)	ND
	Mix peptides 13 + 19	Mix peptides 13 + 19	Mix	Mix
	Mix peptides 18 + 19	Mix peptides 18 + 19	Mix	Mix
	Mix peptides 13 + 47	Mix peptides 13 + 47	Mix	Mix
	Mix peptides 17 + 47	Mix peptides 17 + 47	Mix	Mix
	Mix peptides 19 + 47	Mix peptides 19 + 47	Mix	Mix
Galvani N.C. *et al*., 2021 [26]	KLTSMTPHEFKAICRL	Pept1	Hypothetical protein LiHyT	XP_001465138.1
	RVQATEAQDRDLYARF	Pept2		
	PELYQQYVDYYVMYYE	Pept3	Hypothetical protein LiHyD	XP_001468360.1
	EPLLQQTQRAHMQRQQPAMPQPGYQPPPPM	Pept4		
	SQGASSGTCANAKCIPGNT	Pept5	Hypothetical protein LiHyV	XP_001462854.1
	SSFPITKGAALTVDYGRCE	Pept6		
	EETIRRRHEQRAARVK	Pept7	Hypothetical protein LiHyP	XP_001468385.2
	PRRLAAADLEELASAHEDFVAHLEKAKER	Pept8		
Galvani N.C. *et al*., 2022 [27]	KLTSMTPHEFKAICRL	Pept1	Hypothetical protein LiHyT	XP_001465138.1
	RVQATEAQDRDLYARF	Pept2		
	PELYQQYVDYYVMYYE	Pept3	Hypothetical protein LiHyD	XP_001468360.1
	EPLLQQTQRAHMQRQQPAMPQPGYQPPPPM	Pept4		
	SQGASSGTCANAKCIPGNT	Pept5	Hypothetical protein LiHyV	XP_001462854.1
	SSFPITKGAALTVDYGRCE	Pept6		
	EETIRRRHEQRAARVK	Pept7	Hypothetical protein LiHyP	XP_001468385.2
	PRRLAAADLEELASAHEDFVAHLEKAKER	Pept8		
Gonzales *et al*., 2002 [29]	APKTAKKAAPKDVKATKVVKVT	23083	Ribosomal protein L25	AF131910
	TKVVKVTTRKSYTRPQFRRPHTYRRPAIAKPS	23089		
	RPAIAKPSNRVTESKDITAF	23085		
Link, J.S. *et al*., 2017 [35]	GHRMPPTSVSALARP	P1	GP63	XP_001562922.1
	TMVPKEPNPLSGLRK	P2		
	SKPQPNNFKLNSLGS	P3		
	Mix P1+P2+P3	Mix P1+P2+P3		
Machado, A.S. *et al*., 2020 [37]	KWKTGAALDGAPQPLNTL	PeptC	Hypothetical protein LiHyC	XP_001470432.1
Medeiros *et al*. 2022 [40]	MNEPAPP	peptide	Tryparedoxin peroxidase	LBRM.15.1100
Menezes-Souza, D. *et al*., 2014 [42]	EEDESKKKSCGDEGEPKVE	Peptide 1	HSP83.1	LBRM_33_0340
	VTEGGEDKKK	Peptide 2		
	EVAEAPPAEAAPA	Peptide 3		
Menezes-Souza, D. *et al*., 2015a [43]	VGGGNSKNG	Peptide-1	MAPK3	LBRM_10_0620
	DPAEEADAP	Peptide-2	MAPK4	LBRM_19_1710
Menezes-Souza, D. *et al*., 2015b [41]	QTSGSTTPGPTTTT	Peptide-1	CPB	LBRM_08_0830
Oliveira-da-silva, J.A. *et al*., 2020a [47]	AIREAKQKDHDHSDPTPDKATGSTK	Peptide	Pyridoxal kinase PK	LINJ_30_1310
Oliveira-da-silva, J.A. *et al*., 2020b [48]	EGVQEEDPTSLKNLFV	PeptJ	Hypothetical protein LiHyJ	XP_001462647.1
Ramos F.F. *et al*., 2021 [58]	TSKFWDT	Pep1	Trypoanothione reductase and Tyrosine aminotransferase	2JK6_A and 4IX8_A
	HITANES	Pep2		
	LRINNQS	Pep3	Trypoanothione reductase	2JK6_A
	TTHTYFG	Pep4	Trypoanothione reductase and Glyoxalase II	2JK6_A and 2P18_A
	PAPSRMV	Pep5	Glyoxalase II	2P18_A
	DPSPWRQ	Pep6	Tyrosine aminotransferase	4IX8_A
	HRYSPSF	Pep7	Trypoanothione reductase	2JK6_A
	DPTTQYS	Pep8	ND	ND
	SNYHSRW	Pep9	Tyrosine aminotransferase	4IX8_A
Salles, B.C.S. *et al*., 2017 [62]	FLCSHSN	A3	ND	ND
	TFLFFPA	A5		
	TFLVPLQ	A8		
	RYVSVAS	A11		
	FLSDVGE	B2		
	TFFLRVR	B9		
	INRSIKG	H11		
	LIKISTK	G12		
Salles, B.C.S. *et al*., 2019 [63]	MQKDEESGEFKCEL	Peptide	SMP-3	XP_003873457.1
Vale, D.L. *et al*., 2019 [69]	LPFSISCVFASETRRLARERYGISG	Peptide	Amastin protein	XP_003392700.1
Vale, D.L. *et al*., 2022 [70]	IQFSDSIKRFNELDCE	PepA	Tryparedoxin	XP_001563558.1
	GFSGDSSESYSLSDNSSKVDDRIKL	PepB	Hypothetical protein	XP_001568689.1
	LVSIEDPFAEDNFDEF	PepC	Enolase	XP_001563419.1
	AFRISDPPQYSRVVPA	PepD	Hypothetical protein	XP_001568689.1
	IAKTLRDHGNGRYYLDSDSLYVN	PepE	Prohibitin	XP_001568126.1
	KGDATMKPERQASIE	PepF	Tryparedoxin	XP_001563558.1
	EIGSASKYGYSGWA	PepG	Enolase	XP_001563419.1

The reactivity of all peptides was analyzed with human serum samples. Sample size ranged from 27 to 210 patients (Supplementary data 2). Ten publications assessed VL diagnosis with cases from Brazil (nine studies) [11, 16, 17, 37, 47, 48, 58, 62, 69] and from Sudan (one study) [7]. Four publications assessed TL (CL and ML) diagnosis with cases from Brazil (three studies) [15, 40, 63], and from Peru (one study) [29]. Two publications assessed only CL diagnosis with cases from Brazil (one study) [35] and from Peru (one study) [10]. Finally, six studies assessed peptides diagnosis with TL and VL cases from Brazil in a separate group [26, 27, 41–43] or in the same group [70]. Most of the TL cases studied were due to *L. braziliensis* infection and the VL cases due to *L. infantum*. Five studies did not specify the infecting species [7, 10, 17, 41, 43].

Different reference standards were used to confirm leishmaniasis cases (Table 1). Thirteen studies employed a composite reference test using two methods (parasitological and molecular techniques *n* = 7 [15, 17, 27, 40–43], or parasitological and serological techniques *n* = 1 [7] or serological and molecular techniques *n* = 5 [16, 26, 37, 48, 58]). Two studies employed a composite reference test using three methods (parasitological, molecular and serological techniques) [62, 63]. Seven studies used only one reference standard (parasitological technique *n* = 3 [10, 29, 35] or molecular technique *n* = 4 [11, 47, 69, 70]). No studies used only a commercial serological test as the reference standard.

Several strategies were used to identify peptide candidates in the 22 included articles (Supplementary data 2). Fifteen articles used a bioinformatic method. Five different algorithms were cited: ABCPred [7, 26, 27, 37, 62, 63, 69], BepiPred [7, 11, 17, 40–43], IEDB [26, 27, 47, 70], EpiQuest-B and LBTope [7]. Other strategies for epitope discovery were phage-displayed immunoscreening of random peptides libraries [15, 16, 35, 48, 58] or construction of an overlapping peptide library from the candidate protein sequence [10, 29].

### Performance of peptide-based serological tests

**Figure 2:**
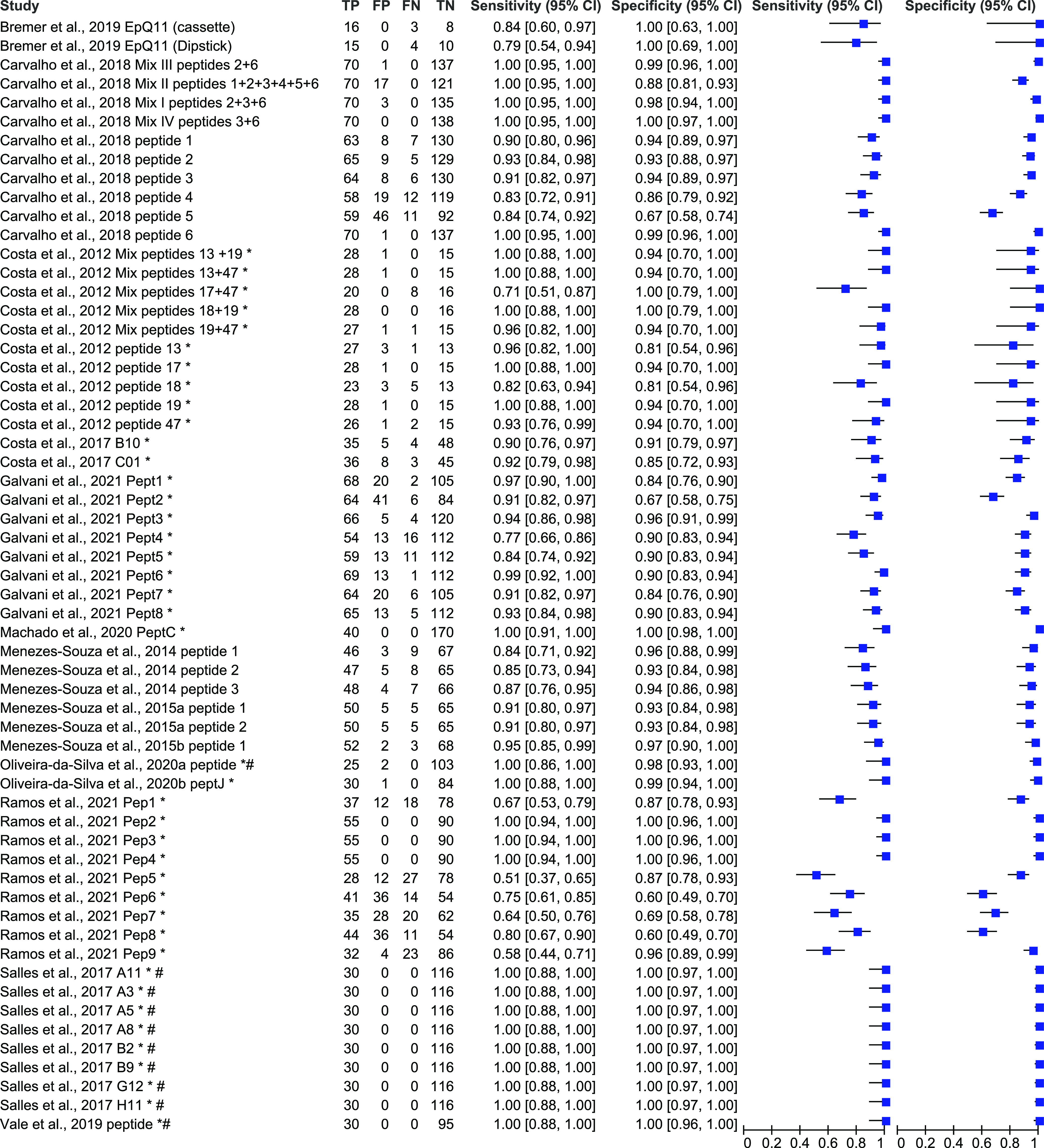
Forest plot representing percentage of sensitivity and specificity of different peptide-based tests for visceral leishmaniasis (VL) diagnosis. * studies where the counts of true positive (TP), false positive (FP), false negative (FN), and true negative (TN) results were estimated from the sensitivity and specificity values. # studies where sensitivity and specificity were recalculated considering all non-cases in a single control group.

**Figure 3:**
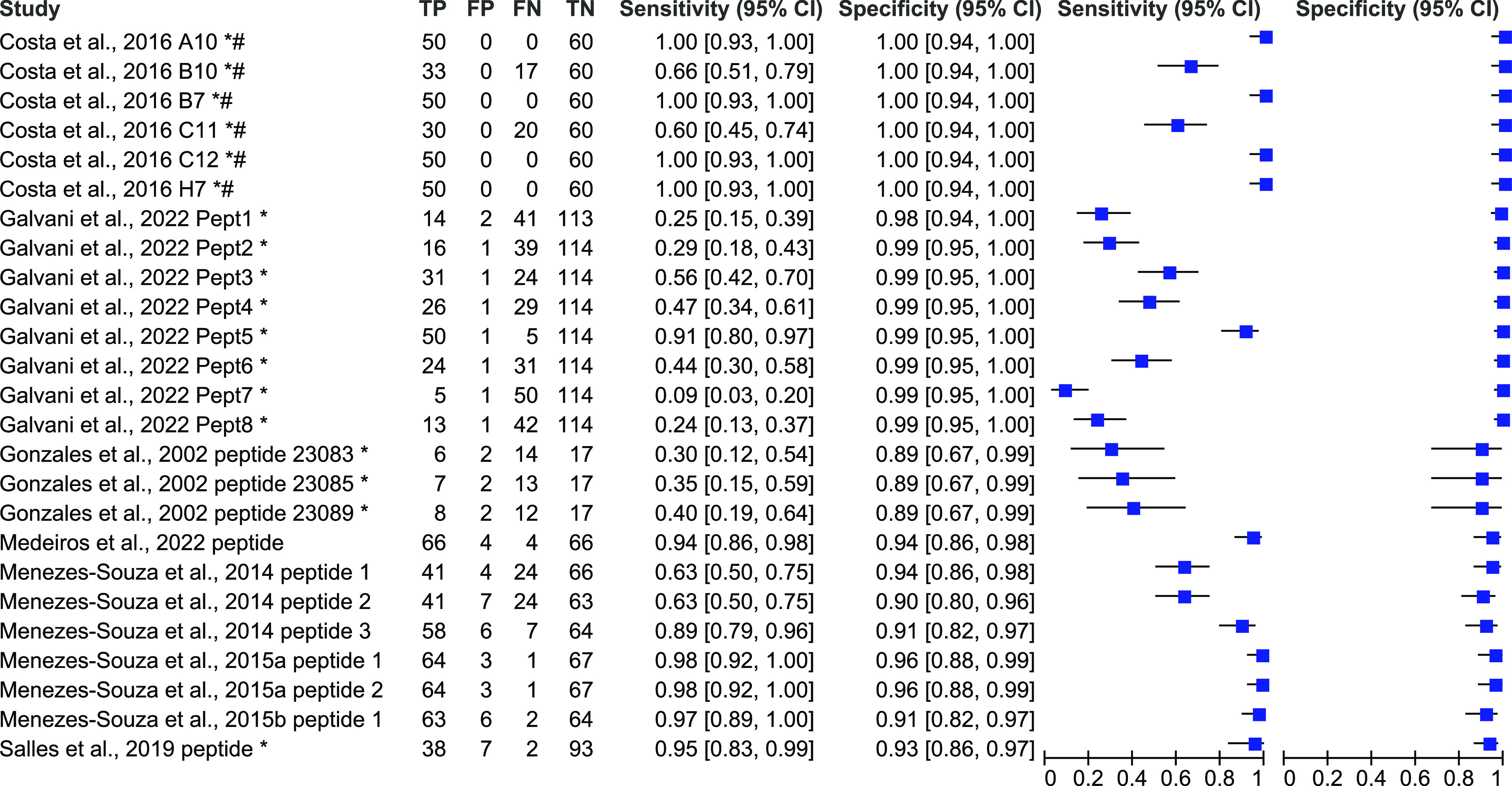
Forest plot representing percentage of sensitivity and specificity of different peptide-based tests for tegumentary leishmaniasis (TL=CL+ML) diagnosis. * studies where the counts of true positive (TP), false positive (FP), false negative (FN), and true negative (TN) results were estimated from the sensitivity and specificity values. # studies where sensitivity and specificity were recalculated considering all non-cases in a single control group.

**Figure 4:**
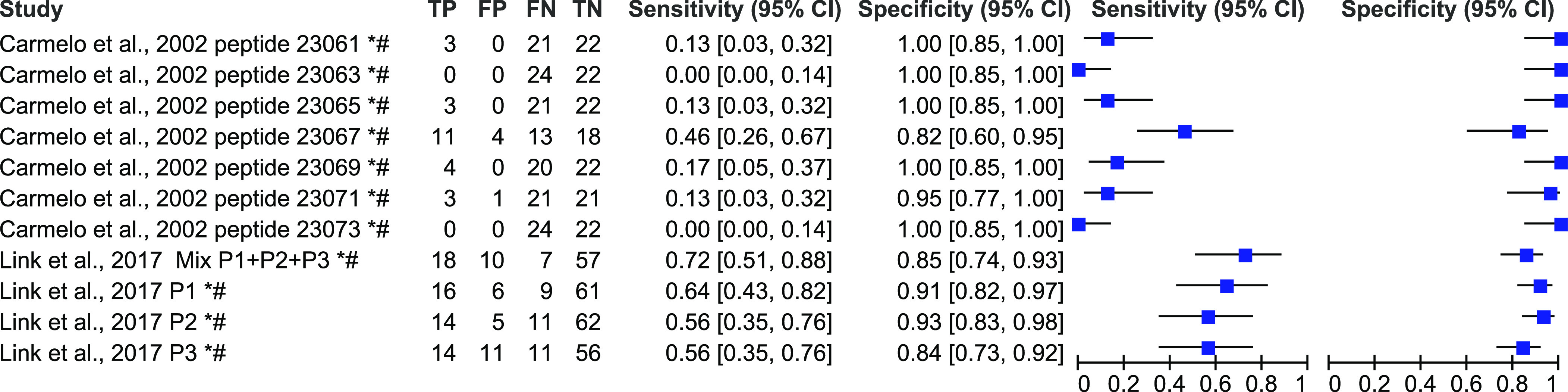
Forest plot representing percentage of sensitivity and specificity of different peptide-based tests for cutaneous leishmaniasis (CL only) diagnosis. * studies where the counts of true positive (TP), false positive (FP), false negative (FN), and true negative (TN) results were estimated from the sensitivity and specificity values. # studies where sensitivity and specificity were recalculated considering all non-cases in a single control group.

**Figure 5:**
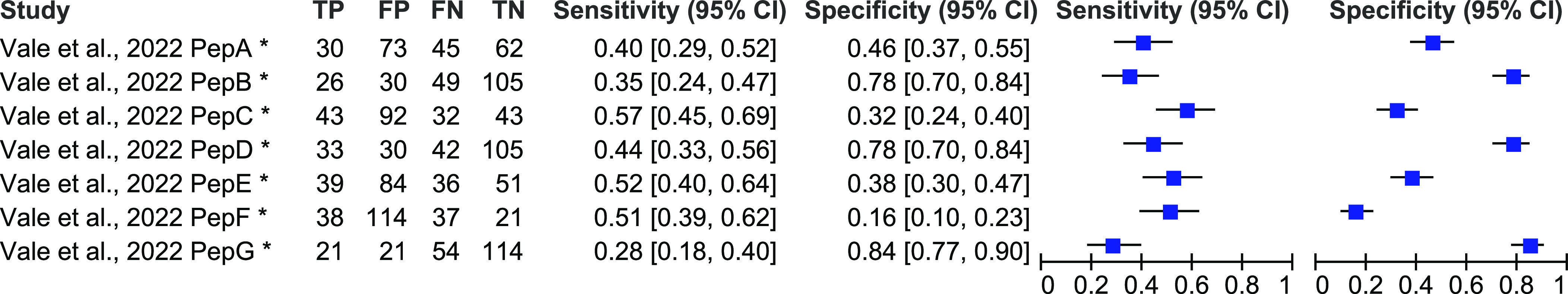
Forest plot representing percentage of sensitivity and specificity of different peptide-based tests for both visceral leishmaniasis (VL) and tegumentary leishmaniasis (TL) diagnosis. * studies where the counts of true positive (TP), false positive (FP), false negative (FN), and true negative (TN) results were estimated from the sensitivity and specificity values. # studies where sensitivity and specificity were recalculated considering all non-cases in a single control group.

Performance of index tests based on different peptides was estimated by assessing sensitivity and specificity. The sensitivity and specificity of peptide-based tests for human VL diagnosis are presented as forest plots in Figure 2, for human TL diagnosis (CL and ML) in Figure 3, for only human CL in Figure 4 and for both VL and TL in Figure 5, based on data extracted from the 22 included articles (Supplementary data 2).

For VL diagnosis, the performance of 49 different synthetic peptides was evaluated from serum of VL patients. Their sensitivity ranges from 51% to 100% and their specificity from 60% to 100% (Fig. 2). Among them, the performance of 17 synthetic peptides was evaluated both in VL patients and VL patients co-infected with HIV. Their sensitivity ranges from 51% to 100% and their specificity from 60% to 100% [26, 58]. High performance with 100% sensitivity and specificity was reported for three peptides [58]. Two studies also evaluated the performance of peptides in combination [11, 17]. The sensitivity of peptide combination ranged from 71% to 100% and specificity from 88% to 100%. Thus, for VL diagnostic, high performance with sensitivity ≥ 95% and specificity ≥ 98% was reported for 16 peptides [11, 37, 47, 48, 58, 62, 69] and four peptide mixtures [11, 17].

For TL diagnosis, the performance of 25 different synthetic peptides was evaluated (Fig. 3). Their sensitivity ranged from 9% to 100%, and specificity from 75% to 100%. High performance with sensitivity ≥ 95% and specificity ≥ 95% was reported for six peptides [15, 43].

For CL diagnosis only, the performance of ten different synthetic peptides was evaluated (Fig. 4). Their sensitivity ranged from 0% to 66%, and specificity from 82 to 100%. One study evaluated the performance of three synthetic peptides in combination [35]. This peptide mixture obtained 77% sensitivity and 85% specificity.

The performance of seven synthetic peptides was evaluated using both VL and TL patients in same group (Fig. 5). Their sensitivity ranged from 28% to 57%, and specificity from 16% to 84% [70].

### Quality assessment of study reports

**Figure 6:**
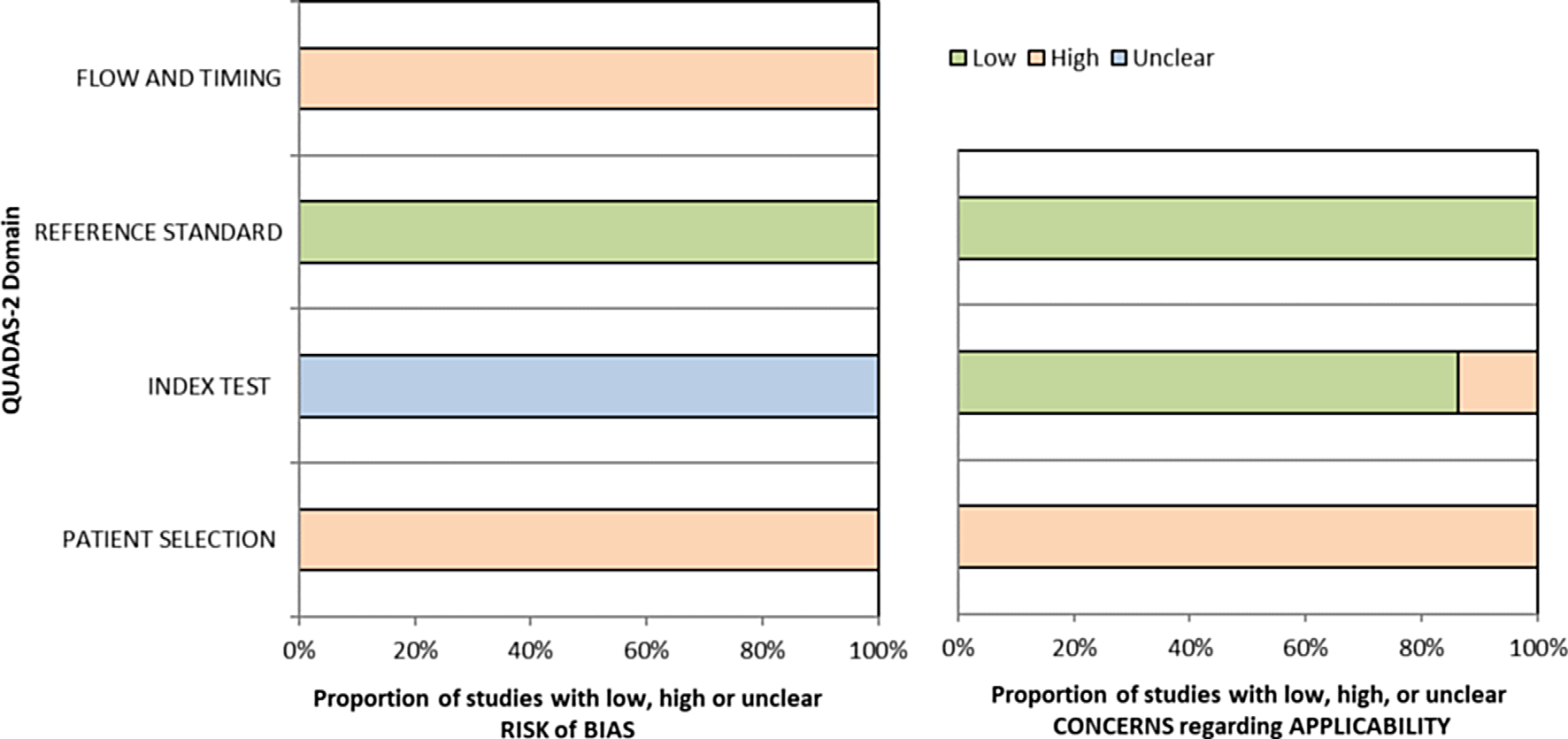
Risk of bias and applicability concerns for the 22 articles included in the review. QUADAS-2 results summarize quality assessment for patient selection, index test, reference standard and flow and timing. This figure was generated using the QUADAS website. (https://www.bristol.ac.uk/population-health-sciences/projects/quadas/quadas-2/).

Although all publications included in this review were in clinical research phase I or II of diagnostic test development, we used the QUADAS-2 tool (more suitable for phase III) to assess the quality of the 22 articles in terms of risk of bias and applicability concerns. The results are summarized in Figure 6 and study details are provided in Supplementary data 3.

All the included studies followed a case-control design with archived or collected samples, selected for convenience and according to the outcome of the reference standard. Thus, they had a high risk of bias and high concern regarding applicability in the domain of patient selection.

No study specified whether the index test was conducted in a double-blind fashion or with random samples, so all studies were classified “unclear” for risk of bias concerning the index test. Three out of 22 studies were at high risk of applicability concerns in the index test domain, because two used prespecified cut-offs based on the control group value (mean with three or two standard deviations to determinate the threshold) instead of using the ROC curve [10, 17], and the third study did not describe how the cut-off value was set [29].

All included studies were evaluated at low risk of bias and applicability concerns regarding the reference standard domain. The reference standard or the composite reference standards used were made before the index test, so the results of reference tests were still interpreted without knowledge of the index tests results.

All included studies in this review were at high risk of bias regarding the domain of flow and timing. In most studies, there were no details on the period and conditions of sample storage or time interval between the performance of index test and the reference standard. However, except for two studies [7, 17], an ELISA test was done to check the presence of antibodies directed against soluble *Leishmania* antigen (SLA) extracted from local *Leishmania* strain. Finally, in studies where the reference standards included a parasitological test, that is considered invasive (lesion biopsies, bone marrow aspirates), the control groups did not receive the same reference standard as patients.

## Discussion and conclusion

### Summary of main results

This literature search allowed a state-of-the-art review of the use of synthetic peptides in serodiagnostics for human leishmaniases from 2002 to 2022. Among included articles, most of them (19/22; 86%) were published in the last ten years, which shows the growing interest in the use of synthetic peptides in leishmaniasis diagnostic tests in the recent years. The development of several algorithms to predict *in silico* linear B-cell epitopes like Bepipred, BCpred, ABCPred and LBtope [24, 59, 65], and the increase in immune epitope data deposited in public databases such as the Immune Epitope Database (IEDB, http://ieddb.org/) [72], promote the surge in the use of synthetic peptides in health research [1]. A combination of bioinformatics tools can be used in the absence of immunoproteomic data from previous research. For example, Carvalho *et al*. used bioinformatics tools in all processes to predict antigenic properties of proteins and to identify B-cell epitopes from this protein. These authors used available data (TritrypDB or SignalP) to identify secreted excreted proteins that are most exposed to the immune system [11]. Furthermore, phage display is also a powerful strategy to rapidly identify disease-specific B-cell epitopes, as has been done for other pathogens, such as those causing severe acute respiratory syndrome (SARS) [36] or dengue fever [68].

The present review reported promising performance with ELISA or phage-ELISA techniques with sensitivity ≥ 95% and specificity ≥ 98% for VL diagnosis, and with sensitivity ≥ 95% and specificity ≥ 95% for TL diagnosis for several peptide-based tests [11, 15, 17, 37, 43, 47, 48, 58, 62, 69]. Only one peptide was assessed in ICT cassette and dipstick formats for which 100% specificity and, 84% and 79% sensitivity were reported, respectively in Sudanese patients [7]. While serological tests are known to have low sensitivity for the diagnosis of VL in HIV-infected compared non-HIV-infected patients [18, 19], two studies reported promising performance (sensitivity from 51% to 100% and specificity from 60% to 100%) of peptide-based test evaluated on VL/HIV co-infected patients [26, 58] of which three peptide-based tests with 100% sensitivity and specificity [58].

Most studies included in this review estimated the performance of peptide-based tests with sera of patients from single geographical areas (Sudan, Brazil or Peru) and infected by a same *Leishmania* species (molecularly identified or assumed), either by *L. infantum* for VL diagnosis or *L. braziliensis* for TL diagnosis. Only six studies, all performed in Brazil, evaluated the performance of the test using both sera of *L. infantum* VL patients and *L. braziliensis* TL patients [26, 27, 41–43, 70].

### Strengths and weaknesses of the review

The review process followed PRISMA guidelines and all included studies were evaluated in accordance with the QUADAS-2 tool. However, the performance data for peptide-based tests should be interpreted with caution because all the studies included in this review are in clinical research phase I or II of diagnostic test development. Thus, the risk of bias for most of these studies was assessed as “unclear” and/or “high” for three of the four domains included in the QUADAS-2 tool, i.e., patient selection, index test, and flow and timing. The main risk of bias in the reviewed studies was related to patient selection. In the first step of diagnostic test development, the inclusion of patients is based on the availability of stored serum samples and on diagnostic information provided by health services or depended on consecutively recruited cases with confirmation using the reference standard. Suspected cases were excluded, leading to overestimation of the diagnostic accuracy of the index test.

The heterogeneity of reference standard and sampling used in the included studies makes it difficult to compare performance results. Accuracy of diagnosis was not the same based on the reference standards used. The parasitological method was used in 59% (13/22) of the studies, but among them, 23% (3/13) used only parasitology as the reference standard [10, 29, 35]. Most other authors have incorporated another technique to confirm the diagnosis such as PCR to detect the kinetoplastid DNA (kDNA) of *Leishmania* parasites (9/13) [15, 17, 27, 40–43, 62, 63] and/or a commercial serological test using the rK39 protein (3/13) [7, 62, 63]. Furthermore, most studies integrated in the control group, in addition to healthy individuals, patients with other diseases such as Chagas disease (18/22), leprosy (8/22), HIV (5/22), malaria (4/22), tuberculosis (4/22), aspergillosis (4/22), paracoccidioidomycosis (3/22), and/or histoplasmosis (2/22), whereas three studies used only healthy individuals in the control group [7, 17, 37]. Also, further heterogeneity was found between studies, and even within some studies, regarding the length and concentration of used peptides, making it difficult to compare the performance results. In studies using the ELISA technique (19/22), peptide concentration was expressed in μg/well or μg/mL, without considering the molecular weight of the peptide, which varies according to the amino acid composition of each peptide. For example, Carvalho *et al*., used 10 μg/well of a 12-amino-acid peptide (peptide 1) that has a theoretical molecular weight of 1274.27 g/mole, whereas for another peptide (peptide 2), they also used 10 μg/well for this 28-amino-acid peptide that has a theoretical molecular weight of 2514.60 g/mole [11]. Therefore, some of the peptides may not have been studied at a saturating molar concentration, which can impact density and immunoreactivity of the immobilized peptides in ELISA microplate wells, and therefore have an influence on test performance [22, 44].

Standardization of the evaluation methodology (in particular the use of a reference standard and the appropriate composition of control group), as well as better knowledge of the QUADAS-2 tool and the STARD statement (Standards for Reporting of Diagnostic Accuracy Studies) [14] by investigators, would be beneficial for the qualities of assessment and the quality report of diagnostic accuracy studies.

### Applicability of findings and implication for practice and research

Given that there are more than 70 endemic countries described with multiple circulating species responsible for different clinical forms of leishmaniasis [81], different treatments depending on the clinical form and the parasite species involved [8], and other diseases that have similar clinical symptoms in same leishmaniasis endemic areas, [31, 57, 66], accurate diagnosis of leishmaniases is an important need. The development of diagnostic tests based on peptides should meet this need. Moreover, synthetic peptides have several other advantages, such as their low cost, simple chemical production, reproducibility, ease of storage, stability and safety [13, 46, 55]. The use of bioinformatics tools helps to easily predict immunodominant epitopes from protein sequences and reduce potentially costly and time-consuming laboratory work. The peptides can be identified from one or several antigens and used in combination to increase the number of reactive epitopes and improve diagnosis performance [9, 23, 45, 56].

This systematic review provides evidence for recommending the use of synthetic peptides for biological serodiagnosis of both TL and VL. Despite promising performances with 100% sensitivity and specificity for several peptide-based tests for VL or TL diagnosis, evaluation on serum samples from patient groups infected by different *Leishmania* species present in a same endemic area is important for accurate diagnosis, such as in Brazil where eight different pathogenic species are present [2]. Importantly, sensitivity can be variable and depend on geographical area, as has already been observed with the rK39-ICT. In VL due to *L. donovani*, rK39-ICT sensitivity was lower in East Africa (85.3%; 95% CI 74.5 to 93.2) than in the Indian subcontinent (97.0%; 95% CI 90.0 to 99.5) [6].

The performance results can be influenced by the accuracy of the reference standards chosen, the composition of control group used, the disease-causing *Leishmania* species of patients’ groups, leishmaniasis clinical form of patient groups, and geographical area of study. The parasitological method, although routinely used in health services for the diagnosis of leishmaniasis, shows low sensitivity [30] and is therefore likely to lead to false-negative results. The results of the parasitological examination may depend on several factors, such as parasitemia or parasite load, the sample, and the skills of the operator for sample collection and microscopic examination. The lack of an accurate gold standard leads to diagnostic errors and can be resolved by using multiple diagnostic methods, such as visualization of amastigote by microscopy, parasite isolation by culture, molecular detection of parasite DNA, and for VL, serological rK39 test [3, 12]. In the evaluation of a new diagnostic test, the use of such a composite reference standard would allow better classification of samples and thus decrease the proportion of false negatives, especially in individuals with low parasitemia.

The specificity assessment of serological diagnostic test for leishmaniasis depends on the differential diagnosis because other diseases with similar clinical manifestation to leishmaniasis are common in endemic areas, but also diseases known to cross-react with anti-*Leishmania* antibodies. The differential diagnosis depends on clinical form and endemic area. For example, it is important to include patients with leprosy and lupus vulgaris for diagnosis of CL, paracoccidioidomycosis and tuberculosis for diagnosis of ML [31, 53], or malaria, typhoid fever, arbovirus diseases (chikungunya, Zika, and dengue fever), acute Chagas disease, acute schistosomiasis, amoebic liver abscess, mononucleosis and hepatitis for diagnosis of acute forms of VL [53]. For example, in the Americas region, many patients with leishmaniasis are co-infected with other tropical diseases [54]. In Brazil, O’Neal *et al*. showed 88% co-infection with helminths in the state of Bahia [49], while Azeredo-Countinho *et al*. diagnosed 15% of patients in the state of Rio de Janeiro [4]. In Argentina, several studies have shown that co-infection with *Trypanosoma cruzi* can be common and the antigenic cross-reactivity between these two parasites makes it difficult to discriminate Leishmaniasis and Chagas disease [25]. As there are a large number of diseases with clinical symptoms similar to those of *Leishmania* infection and cross-reactivities with other parasites, it is pertinent to select the diseases according to the relevant area of co-endemicity to enable proper differential diagnosis.

Therefore, further investigations using large cohorts of cases and non-cases from endemic areas are still required to determine the promising performance of these peptides (alone or in combination with other peptides), and to estimate the accuracy of these peptide-based diagnostic tests in clinical practice. Therefore, peptide-based tests could be very helpful for the development of more efficient point-of-care diagnostic tests, and this regardless of the clinical form of leishmaniasis, the circulating *Leishmania* species and geographical area. Moreover, the synthetic peptides composed of species-specific and conserved epitopes could overcome the problems of specificity and sensitivity found with some antigens and according to geographical regions.

### Protocol and registration

The review protocol was conducted following the guidance of the Preferred Reporting Items for Systematic reviews and Meta-Analyses (PRISMA) statement, PRISMA 2020 statement [51]. The PRISMA 2020 Checklist of the review is provided as supplementary material (Supplementary data 4). The review was not registered and was different from any other review registered or published.

**Abbreviations**
CD: Chagas disease;
CL: Cutaneous leishmaniasis;
CRD: Cross-reactive group;
DAT: Direct agglutination test;
DCL: diffuse cutaneous leishmaniasis;
ELISA: Enzyme linked immunosorbent assay;
HAT: Human African trypanosomiasis;
HE: Healthy individual from endemic area;
HND: Healthy individual from unspecified area endemic or not;
HNE: Healthy individual from non-endemic areas;
ICT: Immunochromatographic test;
IEDB: Immune epitope database and analysis resource;
IFA: Indirect immunofluorescence;
ML: Mucosal or mucocutaneous leishmaniasis;
ND: Not determined or not otherwise specified;
NTDs: Neglected tropical diseases;
PCR: Polymerase chain reaction;
PKDL: Post-kala-azar dermal leishmaniasis;
PRISMA: Preferred reporting items for systematic reviews and meta-analyses;
ROC: Receiver operating characteristic;
Se-CI 95%: Confidence interval of sensitivity;
Sp-CI 95%: Confidence interval of specificity;
SLA: Soluble *Leishmania* antigens;
TL: Tegumentary leishmaniasis;
VL: Visceral leishmaniasis;
WHO: World Health Organization

## Funding

This review received non-financial external support.

## Conflict of interest

The authors declare that they have no conflict of interest.

## Availability of data

All relevant data are included in the review and all the data for all of the tests entered into the review are presented in supplemental data files.

## Supplementary data

The supplementary material of this article is available at https://www.parasite-journal.org/10.1051/parasite/2023011/olm. *Supplementary file 1*: List of references to studies included in this review. *Supplementary file 2*: Characteristics of included studies and peptides. *Supplementary file 3*: Risk of bias and applicability concerns: review authors’ judgements about each domain for each included study. *Supplementary file 4*: PRISMA 2020 Checklist.
